# A data-driven approach to categorize patients with traumatic spinal cord injury: cluster analysis of a multicentre database

**DOI:** 10.3389/fneur.2023.1263291

**Published:** 2023-10-12

**Authors:** Shahin Basiratzadeh, Ramtin Hakimjavadi, Natalie Baddour, Wojtek Michalowski, Herna Viktor, Eugene Wai, Alexandra Stratton, Stephen Kingwell, Jean-Marc Mac-Thiong, Eve C. Tsai, Zhi Wang, Philippe Phan

**Affiliations:** ^1^Telfer School of Management, University of Ottawa, Ottawa, ON, Canada; ^2^Faculty of Medicine, University of Ottawa, Ottawa, ON, Canada; ^3^Department of Mechanical Engineering, Faculty of Engineering, University of Ottawa, Ottawa, ON, Canada; ^4^School of Electrical Engineering and Computer Science, Faculty of Engineering, University of Ottawa, Ottawa, ON, Canada; ^5^Division of Orthopedic Surgery, Ottawa Hospital Research Institute (OHRI), Ottawa, ON, Canada; ^6^Department of Surgery, Faculty of Medicine, University of Ottawa, Ottawa, ON, Canada; ^7^Hôpital du Sacré-Cœur de Montréal, Montreal, QC, Canada; ^8^Faculty of Medicine, University of Montreal, Montreal, QC, Canada; ^9^Division of Neurosurgery, The Ottawa Hospital, Ottawa, ON, Canada; ^10^Department of Cellular and Molecular Medicine, Faculty of Medicine, University of Ottawa, Ottawa, ON, Canada; ^11^Department of Orthopedic Surgery, University of Montreal Health Center, Montreal, QC, Canada

**Keywords:** traumatic spinal cord injury, patient-centric approach, patient categorization, data-driven method, cluster analysis

## Abstract

**Background:**

Conducting clinical trials for traumatic spinal cord injury (tSCI) presents challenges due to patient heterogeneity. Identifying clinically similar subgroups using patient demographics and baseline injury characteristics could lead to better patient-centered care and integrated care delivery.

**Purpose:**

We sought to (1) apply an unsupervised machine learning approach of cluster analysis to identify subgroups of tSCI patients using patient demographics and injury characteristics at baseline, (2) to find clinical similarity within subgroups using etiological variables and outcome variables, and (3) to create multi-dimensional labels for categorizing patients.

**Study design:**

Retrospective analysis using prospectively collected data from a large national multicenter SCI registry.

**Methods:**

A method of spectral clustering was used to identify patient subgroups based on the following baseline variables collected since admission until rehabilitation: location of the injury, severity of the injury, Functional Independence Measure (FIM) motor, and demographic data (age, and body mass index). The FIM motor score, the FIM motor score change, and the total length of stay were assessed on the subgroups as outcome variables at discharge to establish the clinical similarity of the patients within derived subgroups. Furthermore, we discussed the relevance of the identified subgroups based on the etiological variables (energy and mechanism of injury) and compared them with the literature. Our study also employed a qualitative approach to systematically describe the identified subgroups, crafting multi-dimensional labels to highlight distinguishing factors and patient-focused insights.

**Results:**

Data on 334 tSCI patients from the Rick Hansen Spinal Cord Injury Registry was analyzed. Five significantly different subgroups were identified (*p*-value ≤0.05) based on baseline variables. Outcome variables at discharge superimposed on these subgroups had statistically different values between them (*p*-value ≤0.05) and supported the notion of clinical similarity of patients within each subgroup.

**Conclusion:**

Utilizing cluster analysis, we identified five clinically similar subgroups of tSCI patients at baseline, yielding statistically significant inter-group differences in clinical outcomes. These subgroups offer a novel, data-driven categorization of tSCI patients which aligns with their demographics and injury characteristics. As it also correlates with traditional tSCI classifications, this categorization could lead to improved personalized patient-centered care.

## 1. Introduction

Traumatic spinal cord injury (tSCI) has significant physical, social, and vocational consequences for patients and their families (1). The loss of independence, increased lifelong mortality rates, and high costs for care place a great burden on the individuals and the healthcare system (1–3), making the appropriate treatment of this devastating disorder crucially important. The management of tSCI requires significant health care resource utilization (4), owing to a possible need for short-term intensive acute care and appropriate management of long-term secondary complications (3). Better specialization for managing tSCI is needed to address the unique needs of patients and to better allocate healthcare resources (5). The implementation of targeted care and effective treatment options could produce substantial benefits for both the patient and the healthcare system.

Early research suggests that specialized care, as opposed to general care, can help produce positive outcomes, including decreased length of stay (LOS) and decreased incidence of secondary complications (6–8). However, the optimal model of healthcare delivery for patients with tSCI has not yet been defined (5); despite current advances, the considerable heterogeneity within the tSCI patient population remains a prominent challenge (9). The variety of pathologies, levels of neurological impairment, and different potentials for recovery within tSCI patients (10, 11) makes it difficult to determine the efficacy of management strategies when novel therapies and standards of care are applied to a group with mixed needs and outcome trajectories. The identification of tSCI patient subgroups with clinically similar characteristics should facilitate better communication between patients and providers, guide optimal management, and inform the development of targeted therapies and models of care.

The categorization and management of tSCI have traditionally been guided by established classifications. Most tSCI studies rely on the International Standards for the Neurological Classification of Spinal Cord Injury (ISNCSCI) to classify patients into groups, which is considered the gold standard for neurological assessment (12, 13). Based on the ISNCSCI, the American Spinal Injury Association Impairment Scale (AIS) is a measure of the neurological severity of injury, and is the most important predictor of recovery in tSCI patients (14). However, classification based on AIS grade alone does not adequately address the heterogeneity observed in the tSCI population; there is considerable variation in spontaneous recovery within each AIS grade (range: A–D), leading to differences in recovery trajectories between patients with presumedly similar initial clinical impairment (11). In other words, knowledge about individual prognostic variables for tSCI provides limited information about complex interactions between other variables and how they may influence prognosis. While the AIS grade itself might be the most important indicator for the prediction of recovery, other clinical factors such as age, injury characteristics, and functional measures have also been reported as significant prognostic variables (15).

As a first step towards understanding the heterogeneity inherent in tSCI, Dvorak and colleagues (9) proposed a classification scheme based on the joint use of baseline neurological level of injury (NLI) and severity of neurological impairment (i.e., AIS grade) – two of the predominant predictors of neurological outcome (13, 15). This approach was deemed the “Canadian Classification” and serves to guide tSCI researchers on how to better classify patients for clinical trials, and how to avoid unrecognized heterogeneity (or imbalances) between treatment groups. Dvorak’s work made several important contributions, including a demonstration that classifying based on the joint distribution of the two baseline characteristics (level and severity of injury), beyond simple univariable classification, can reveal meaningful differences in the recovery potential of patients (9).

The digital age has produced a wealth of healthcare data, providing new opportunities to apply data analytics for improved decision-making by facilitating predictive modeling, treatment pattern identification, and detection of subtle correlations that may be overlooked in traditional methods (16, 17).

Through the lens of data analytics, we aim to build upon previous research by using unsupervised machine learning and specifically spectral clustering (SC), to examine the simultaneous interactions within multiple variables and identify previously unrecognized associations in a data-driven manner. In this approach, the analysis is based on the data itself rather than being influenced by preconceived notions or assumptions about the data (17, 18). Our study therefore represents a data-driven approach to understanding and categorizing tSCI that could potentially guide management of these complex injuries. Notably, such a methodology has previously been applied to research on adult spinal deformity (19).

We hypothesize that a data-driven approach can identify subgroups with clinical similarity within a heterogeneous population of tSCI patients and provide a clinically relevant categorization. To this end, the objectives of this study are to (1) apply an unsupervised machine learning approach, specifically cluster analysis, to identify subgroups of tSCI patients using patient demographics and injury characteristics at baseline, (2) to find clinical similarity within subgroups using etiological variables and outcome variables at discharge, and (3) to categorize patients with clinically similar characteristics by creating multi-dimensional labels.

## 2. Methods

This was a retrospective study using prospectively collected data from a large national, multicenter SCI registry. It included variables from different time points (e.g., admission, inpatient rehabilitation, and discharge), and was conducted in two phases.

During the first phase, SC was performed on a subset of variables at baseline to identify subgroups of tSCI patients. Clinical similarities were then identified between each subgroup by superimposing the outcome variables at discharge and etiological variables. The rationale behind exclusively forming subgroups based on baseline variables, and then superimposing outcome variables, is to assess the distinction among patient categories, with respect to selected outcomes. This choice protocol results in a subgrouping independent of outcome variables. As such, the identified subgroups can later be studied against a range of outcomes.

During the second phase, the results were interpreted from a point of view of statistical significance between each group. Thereafter, exemplars were used to describe (or “label”) patient’s subgroups qualitatively and systematically to reveal any patient-centred insights that can be drawn from the identified clinically similar subgroups.

### 2.1. Rick Hansen Spinal Cord Injury Registry

The analyzed data set consisted of patients enrolled in the Rick Hansen Spinal Cord Injury Registry (RHSCIR): a Canada-wide, prospectively collected multicenter database (20). RHSCIR collects data on individuals who have sustained an acute tSCI and received care at one of the participating 18 acute or 12 rehabilitation sites. All sites obtained Institutional Research Ethics Board (REB) approval to enroll patients and enter their data into the registry. A wide variety of data was collected from the pre-hospital, surgical, acute, and in-patient rehabilitation phases of the enrolled patients’ care, including but not limited to: socio-demographic factors, medical history, injury details, diagnoses and interventions, neurologic impairment, complications, and patient-reported outcomes. Upon discharge, a survey was conducted at 1, 2, 5, and 10-year intervals (from the date of injury) to obtain patient-reported outcome measures. The registry was created to support research and facilitate the implementation and optimization of best clinical practices (20).

### 2.2. Study population

The data for this study was collected from RHSCIR, which enrolled 8,273 patients with acute tSCI between 2004 and 2017. Data were extracted from RHSCIR for all eligible patients with an acute tSCI between the year 2004 to 2017. Patients were included in this study according to the following inclusion criteria:

- The potential participant was at least 18 years old at the time of injury.
- They had complete data for the variables of interest collected at the acute (0–15 days) time stamp.
- They had complete data for the variables of interest collected at discharge.
- They provided explicit consent for specific data collection, including patient-reported outcomes, across all study time points.

**Figure 1:**
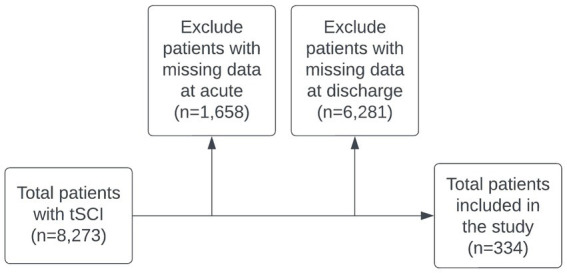
Study population flowchart. Noted that 4,164 patient cases labeled as ‘missing data’ resulted from the absence of explicit consent across all study time points.

Of the original data, 334 patients met the inclusion criteria for the study. The specific numbers of patients adhering to these criteria at each stage of the study are shown in the flowchart in Figure 1.

While the tSCI patient population is the focus of the present study, we note that all spine trauma patients with injuries from the C1 to L5 vertebrae were considered. The spinal cord terminates at the conus medullaris, most commonly at the L1 vertebral body or L1-2 disk interspace level in adults (21). Injuries to the lumbar vertebral bodies may involve the lumbosacral nerve roots and cause cauda equina syndrome (CES), which is not strictly a type of SCI. We chose to include patients with injuries to all levels of the spine and observe the patterns that emerge from the data. Thus, our population of interest includes all patients with impairment of the spinal cord or cauda equina function resulting from trauma.

### 2.3. Variable selection

Variables related to patient demographic, injury, outcome characteristics, and etiology of tSCI were selected based on supporting clinical literature (1, 3, 15, 22–28), expert opinion, and availability in RHSCIR database. The list of variables selected from the dataset are presented in Table 1. Note that the neurological assessments included in the dataset were conducted within a time frame of less than 15 days following the injury.

**Table 1:** The list of baseline, outcome, and etiological variables selected in our study.

Variable name	Description	Variable type	Values
a. Baseline variables			
Age	At the time of injury	Numerical	Age (years)
Body Mass Index (BMI)	BMI is measured in kg/m^2^. Dichotomized to obese (BMI ≥ 30) or not obese (BMI < 30).	Categorical	Obese, not_obese
Baseline AIS class	The severity of neurological impairment collected at admission. Range: a (severe, motor and sensory complete injury) to D (motor and sensory incomplete injury). AIS E is normal.	Categorical	AIS acute A, AIS acute B, AIS acute C, AIS acute D
Primary Location of Injury (PLI)	The vertebral level in the spinal column where the trauma occurred. Range: C1-L5	Categorical	C1,C2,C3,C4,C5,C6,C7, T1,T2,T3,T4,T5,T6,T7, T8,T9,T10,T11,T12,L1, L2,L3,L4,L5
Baseline FIM motor score	An examination of global motor function collected at admission to rehabilitation.	Numerical	FIMMotorScore_adm [13–91]
b. Outcome variables			
FIM motor score at discharge	An examination of global motor function collected at discharge from rehabilitation.	Numerical	FIMMotorScore_disch [13–91]
FIM motor score change	The difference between discharge and admission FIM motor scores	Numerical	Fim Motor Difference
Length of stay	The total length of stay in days, from the time of admission at the hospital to community discharge	Numerical	LOSTotal (days)
c. Etiological variables			
Energy	The energy of the mechanism of the injury	Categorical	Energy_High, Energy Low
Mechanism of injury	The initial mechanical force delivery to the spinal cord and cause the injury	Categorical	Injury_Transport, Injury_Assault – blunt, Injury_Assault – penetrating, Injury_Fall, Injury_Other traumatic cause, Injury_Sports

#### 2.3.1. Outcome variables selection

Both patients and the healthcare system stand to benefit from more specialized tSCI care (5, 29). While the optimal method of healthcare delivery for this patient population has yet to be fully elucidated, there is emerging evidence that specialized care for tSCI patients is associated with reduced LOS and decreased overall mortality (5). The clustering used in this study, driven by baseline patient- and injury-related characteristics, should itself be able to produce clinically similar subgroups with distinct healthcare needs. In this case, patient needs were evaluated using the total Functional Independence Measure (FIM) motor score at discharge (a prognostic factor for long-term outcomes and economic burden) (30), FIM motor score change (measured as the difference between discharge and admission FIM motor scores), and the total LOS (a surrogate measure for healthcare resource utilization).

#### 2.3.2. Etiological variables

In this study, “etiological variables” refers to factors contributing to the cause or origin of the traumatic spinal cord injuries. Our analysis incorporated these variables with the aim of capturing crucial aspects of the injury’s cause and initial impact. These aspects can influence the severity of the injury and the patient’s subsequent recovery.

Two etiological variables were included in our study: “Mechanism of Injury,” and “Energy.” “Mechanism of Injury” describes the initial mechanical force that caused the SCI, such as a fall, a motor vehicle accident, and other types of incidents. “Energy” refers to the intensity of the initial mechanical force that led to a patient’s injury (15, 22).

### 2.4. Cluster analysis and interpretation methods

Cluster analysis (CA) is an unsupervised learning method used for revealing hidden structures in data. Cluster analysis group objects with similar traits into subgroups while minimizing intragroup heterogeneity (16, 18, 31). To find the subgroups of tSCI patients based on baseline variables in the present study (Table 1), multiple clustering algorithms were assessed, and SC was chosen to explore patterns in our data. SC varies from the most commonly used partitioning-based clustering algorithm, K-means clustering (32, 33) as it is not dependent on distance from a centroid and it also does not require clusters to be spherical. Rather, the distance metric used for SC ensures that cluster members are near to one another. As a result, patients in the same subgroups tend to have similar values based on selected characteristics. Unlike K-means, which identifies subgroups with linear borders, SC is capable of forming subgroups with nonlinear boundaries (34).

To identify the optimal number of subgroups, the performance of SC using different numbers of subgroups was evaluated using the Silhouette Coefficient, the Davies-Bouldin index, and the elbow method (35–37).

### 2.5. Interpretation methods

Statistically significant differences and exemplars were used to interpret key findings of the analysis.

#### 2.5.1. Statistically significant difference

After subgroups were created using SC, continuous variables were analyzed using a Kruskal-Wallis test (not meeting the normality assumption) (38) to identify whether there were statistically significant intergroup differences across all subgroups (considered as *p* ≤ 0.05). Although the *p*-value is not traditionally a focal point in cluster analysis, its application here provided deeper insights by enabling us to identify distinguishing factors among subgroups. Variables found to statistically differ were then explored in a pairwise manner between subgroups using Mann–Whitney U tests (39), further enhancing our understanding of the patient subgroups. Continuous variables were presented with means and standard deviations, whereas categorical variables were presented with frequency of occurrence and percentages.

#### 2.5.2. Exemplars

The concept of exemplars is used in this study to provide a tangible illustration of the characteristics of each identified cluster. Exemplar terms are used to represent each subgroup, and are also defined as the medoid of each cluster. Medoids are known as “actual objects” in the data (i.e., a typical patient); the object within a cluster that has the minimum sum of distances to all other objects in the cluster. Therefore, typical patient cases from each subgroup have been provided as a method of interpretation of SC analysis. Using this method, significant findings from statistically significant tests can be demonstrated for each subgroup.

#### 2.5.3. Subgroup labelling

Once patient subgroups were identified using SC analysis, they were then qualitatively described (i.e., “labelled”) to elucidate patient-centred insights that could be learned from the data-driven groupings of patients. Three types of multi-dimensional labels were created to demonstrate the clinical similarity of the following characteristics: (1) patient at presentation, (2) spine injury, and (3) patient at discharge. These multi-dimensional labels served to highlight the distinguishing factors that emerged in each patient subgroup with similar characteristics and to explore whether the “label” (i.e., the pattern of values of distinguishing variables in each subgroup) provided clinically intuitive and plausible characterization in the context of tSCI. Labelling was approached systematically; for each subgroup, only those variables that were determined to have values statistically different from other subgroups were considered as “distinguishing” and thus were used as labels. In addition, we discussed the relevance of the identified subgroups based on the etiological variables and compared them with the literature.

## 3. Results

The 334 patients that met the inclusion criteria for the study and were divided into 5 subgroups using SC. These subgroups were verified as clinically relevant through consultation with field experts and literature review. Table 2 shows the variables at baseline that were found to have statistically significant differences (*p* < 0.05) between the subgroups. Tables 3, 4 present the outcome variables and additional variables related to the mechanism of injury superimposed on each subgroup.

**Table 2:** Baseline variables.

Variable	*P*-value (among all subgroups)	Subgroup 1	Subgroup 2	Subgroup 3	Subgroup 4	Subgroup 5
*N* (%)						
PLI						
C1	<0.001	2 (1.77)	1 (1.39)	0 (0.0)	0 (0.0)	0 (0.0)
C2	<0.001	9 (7.96)*b	0 (0.0)	0 (0.0)	0 (0.0)	0 (0.0)
C3	<0.001	48 (42.48)*a	0 (0.0)	0 (0.0)	0 (0.0)	0 (0.0)
C4	<0.001	66 (58.41)*a	1 (1.39)	0 (0.0)	0 (0.0)	0 (0.0)
C5	<0.001	68 (60.18)*a	22 (30.56)*a	0 (0.0)	0 (0.0)	0 (0.0)
C6	<0.001	52 (46.02)*b	39 (54.17)*b	0 (0.0)	0 (0.0)	0 (0.0)
C7	<0.001	29 (25.66)*a	30 (41.67)*a	0 (0.0)	0 (0.0)	0 (0.0)
T1	<0.001	1 (0.88)	3 (4.17)	0 (0.0)	0 (0.0)	0 (0.0)
T2	<0.001	1 (0.88)	0 (0.0)	4 (10.0)*a	0 (0.0)	0 (0.0)
T3	<0.001	0 (0.0)	0 (0.0)	10 (25.0)*a	0 (0.0)	0 (0.0)
T4	<0.001	0 (0.0)	0 (0.0)	19 (47.5)*a	0 (0.0)	0 (0.0)
T5	<0.001	0 (0.0)	0 (0.0)	17 (42.5)*a	0 (0.0)	0 (0.0)
T6	<0.001	0 (0.0)	0 (0.0)	15 (37.5)*a	0 (0.0)	0 (0.0)
T7	<0.001	0 (0.0)	4 (5.56)*b	8 (20.0)*a	0 (0.0)	0 (0.0)
T8	<0.001	0 (0.0)	9 (12.5)*a	0 (0.0)	0 (0.0)	0 (0.0)
T9	<0.001	0 (0.0)	11 (15.28)*b	0 (0.0)*c	4 (16.0)*b	0 (0.0)
T10	<0.001	0 (0.0)	0 (0.0)	0 (0.0)	10 (40.0)*a	0 (0.0)
T11	<0.001	0 (0.0)	0 (0.0)	1 (2.5)*c	21 (84.0)*a	0 (0.0)
T12	<0.001	0 (0.0)	0 (0.0)	1 (2.5)*b	12 (48.0)*b	28 (33.33)*b
L1	<0.001	0 (0.0)	0 (0.0)	1 (2.5)*c	0 (0.0)	48 (57.14)*a
L2	<0.001	0 (0.0)	0 (0.0)	0 (0.0)	0 (0.0)	12 (14.29)*a
L3	<0.001	0 (0.0)	0 (0.0)	0 (0.0)	0 (0.0)	11 (13.1)*a
L4	<0.001	0 (0.0)	0 (0.0)	0 (0.0)	0 (0.0)	4 (4.76)*c
L5	<0.001	0 (0.0)	0 (0.0)	0 (0.0)	0 (0.0)	4 (4.76)*c
AIS acute D	3.04E-16	65 (57.52)*a	2 (2.78)*c	2 (5.0)*c	2 (8.0)*c	28 (33.33)*a
AIS acute C	0.00021	35 (30.97)*b	5 (6.94)*c	2 (5.0)*c	4 (16.0)	16 (19.05)*b
AIS acute B	1.02E-05	4 (3.54)*b	22 (30.56)*b	4 (10.0)	4 (16.0)	14 (16.67)*c
AIS acute A	8.14E-18	9 (7.96)*a	43 (59.72)*b	32 (80.0)*a	15 (60.0)*b	26 (30.95)*a
Obese patients	0.002	49 (43.36)*c	29 (40.28)*c	29 (72.5)*b	18 (72.0)*b	39 (46.43)*c
Mean (standard deviation)						
Age	1.02E-11	54.15 (16.27)*a	40.22 (15.94)	38.12 (15.31)	43.0 (16.14)*c	36.7 (16.13)*c
FIMMotorScore_adm	4.47E-19	29.07 (20.49)*b	21.6 (9.46)*b	31.55 (12.53)*a	35.0 (12.05)*a	44.71 (15.24)*a
Number of patients	NA	113	72	40	25	84

*a: significantly different from all 4 other subgroups; *b: significantly different from 3 other subgroups; *c: significantly different from 2 other subgroups. All variables are significantly different across subgroups at *p* < 0.00 significance level using Kruskal-Wallis.

**Table 3:** Outcome variables superimposed on identified subgroups.

Variable	*p*-value (among all subgroups)	Subgroup 1	Subgroup 2	Subgroup 3	Subgroup 4	Subgroup 5
Mean (standard deviation)						
LOSTotal	9.99E-13	117.94 (58.4)*c	182.01 (85.31)*a	132.2 (61.57)*b	99.44 (43.69)*c	92.77 (43.75)*b
FIMMotorScore_disch	4.70E-12	63.45 (25.67)*c	50.29 (24.12)*a	64.3 (17.27)*b	74.12 (15.13)*b	79.21 (7.58)*a
Fim motor difference	0.09	34.38 (21.01)	28.69 (20.29)	32.75 (16.45)	39.12 (14.18)	34.5 (14.98)

*a: significantly different from all 4 other subgroups; *b: significantly different from 3 other subgroups; *c: significantly different from 2 other subgroups. All variables are significantly different across subgroups at *p* < 0.00 significance level using Kruskal-Wallis.

**Table 4:** Etiological variables superimposed on identified subgroups.

Etiological variables	Subgroup 1	Subgroup 2	Subgroup 3	Subgroup 4	Subgroup 5
Energy_High (%)	21 (18.58)	38 (52.78)	34 (85.0)	12 (48.0)	49 (58.33)
Energy_Low (%)	86 (76.11)	34 (47.22)	5 (12.5)	13 (52.0)	30 (35.71)
Number of Injury_Transport (%)	22 (19.47)	26 (36.11)	17 (42.5)	9 (36.0)	31 (36.9)
Number of Injury_Assault – blunt (%)	3 (2.65)	1 (1.39)	0 (0.0)	0 (0.0)	0 (0.0)
Number of Injury_Assault – penetrating (%)	2 (1.77)	2 (2.78)	2 (5.0)	0 (0.0)	3 (3.57)
Number of Injury_Fall (%)	64 (56.64)	24 (33.33)	11 (27.5)	12 (48.0)	30 (35.71)
Number of Injury_Other traumatic cause (%)	7 (6.19)	6 (8.33)	3 (7.5)	2 (8.0)	8 (9.52)
Number of Injury_Sports (%)	15 (13.27)	13 (18.06)	7 (17.5)	2 (8.0)	12 (14.29)

### 3.1. Subgroup labels and exemplars

**Figure 2:**
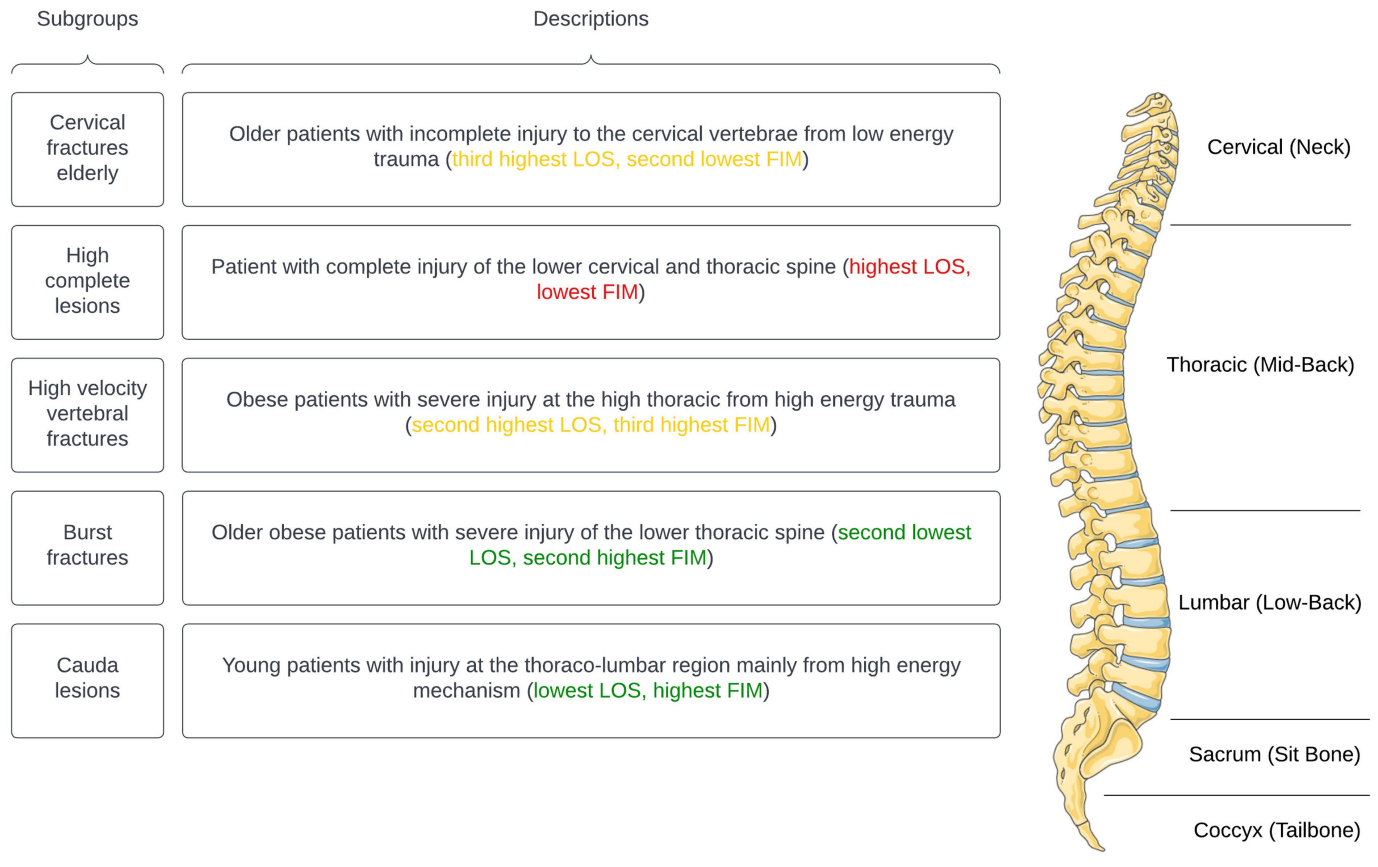
Subgroups with clinically significant phenotypes (parts of the figure were drawn by using pictures from Servier Medical Art. Servier Medical Art by Servier is licensed under a Creative Commons Attribution 3.0 Unported License).

Multi-dimensional labels were created to describe patient demographic and injury characteristics with distinguishing variables at baseline and discharge. Where applicable, specifiers were used to describe variables on a spectrum of severity (i.e., “mild,” “moderate,” “severe” or “extreme”) relative to other subgroups (Table 5). Additionally, a phenotype anatomical figure was constructed to visually highlight the anatomical order of the subgroups, providing a perspective on patient demographics and injury traits (Figure 2). Lastly, exemplars (typical patient case representative of each subgroup) were described.

**Table 5:** Multi-dimensional labels with distinguishing variables for identified subgroups.

Subgroup/label	Patient at presentation	Spine injury	Patient at discharge
Subgroup 1	Older non-obese patient with moderate functional impairment	Motor incomplete cervical injury from low energy fall	Moderate: third highest mean LOS, second lowest mean FIM motor score at discharge
Subgroup 2	Non-obese patient with severe functional impairment	Motor complete cervical or/and thoracic injury from diverse mechanisms	Poor: highest mean LOS, lowest mean FIM motor score at discharge
Subgroup 3	Obese patient with moderate functional impairment	Complete (severe) thoracic injury from high energy motor vehicle accident	Moderate: second highest mean LOS, third highest mean FIM motor score at discharge
Subgroup 4	Obese, older patient with mild functional impairment	Complete (severe) lower thoracic injury from diverse mechanisms	Moderate: second lowest mean LOS, second highest mean FIM motor score at discharge
Subgroup 5	Young patient with mild functional impairment	Bimodal (severe and non-severe) lumbar injury from diverse mechanisms	Favourable: lowest mean LOS, highest mean FIM motor score at discharge

#### 3.1.1. Exemplar for subgroup 1

60-year-old non-obese individual presenting with a motor incomplete injury (AIS D) to the C3-C4 vertebrae, an NLI at C4, and a FIM motor score of 35 at admission. Patient 1’s total length of stay was 102 days before being discharged with a FIM motor score of 87.

#### 3.1.2. Exemplar for subgroup 2

37-year-old non-obese individual presenting with a motor and sensory complete injury to the C6-C7 vertebrae, an NLI of C5, and a FIM motor score of 24 at admission. Patient 2’s total length of stay was 212 days before being discharged with a FIM motor score of 75.

#### 3.1.3. Exemplar for subgroup 3

36-year-old non-obese individual presenting with a motor and sensory complete injury to the T4-T5 vertebrae, an NLI at T2, and a FIM motor score of 30 at admission. Patient 3’s total length of stay was 112 days before being discharged with a FIM motor score of 73.

#### 3.1.4. Exemplar for subgroup 4

47-year-old obese individual presenting with a motor and sensory complete injury to the T11-T12 vertebrae, and NLI at L2, and a FIM motor score of 49 at admission. Patient 4’s total length of stay was 101 days before being discharged with a FIM motor score of 84.

#### 3.1.5. Exemplar for subgroup 5

26-year-old non-obese individual presenting with a motor incomplete (AIS D) to the L1 vertebra, an NLI at L2, and a FIM motor score of 50 at admission. Patient 5’s total length of stay was 105 days before being discharged with a FIM motor score of 88.

## 4. Discussion

In the present study, an unsupervised machine learning approach of cluster analysis utilizing SC was deployed to categorize tSCI patients into clinically similar subgroups based on patient demographics and injury characteristics at baseline. Clustering based on baseline data enabled an exploration for latent relationships between patient demographic and injury characteristics without depending on selected outcomes that might not be comprehensive measures of the condition’ behaviour.

The five patient subgroups with clinical similarities were defined with a data-driven approach that did not rely on *a priori* assumptions or outcome variables, and subgroups were distinguished based on age, BMI, baseline injury severity (AIS grade), PLI, and baseline FIM motor score. The three outcome variables used in the study (FIM motor score at discharge, FIM motor score change, and total LOS) were superimposed on these subgroups to evaluate the distinction of patient subgroups and to explore their clinical relevance. FIM motor score at discharge and total LOS resulted in statistically significant differences across all patient subgroups (Table 3).

The choice of input variables in cluster analysis is a crucial factor that can influence the quality of the analysis and, by extension, the robustness of the conclusions drawn from the study. As part of our research, we carefully selected baseline variables based on their relevance, clinical significance, and prior evidence of impact on patient outcomes (Table 1). This choice ensured that our cluster analysis was grounded in a strong theoretical and empirical basis. Several studies have demonstrated the importance of these variables in patient outcomes. For instance, age plays a significant role in recovery and functional outcomes following tSCI (1, 3). Similarly, the AIS classification at baseline has shown to be a strong predictor of neurological recovery and rehabilitation outcomes (25). The baseline FIM motor score has been identified as a valuable predictor for functional outcomes and discharge planning in tSCI patients (27). While both AIS scores and the total FIM motor score provide insights into a patient’s neurological and functional statuses, their combined use in our research presents a holistic, patient-centered approach. The AIS details specific neurological information, while the total FIM motor score measures patient-reported motor functionality. Together, they furnish a comprehensive understanding of the patient’s condition and experience. Lastly, the primary location of injury (PLI) has been linked to variations in functional outcomes and recovery potential (26).

Though obesity is not a prognostic variable commonly considered in the tSCI patient population (15), it was included in the present study based on several sources of supporting evidence. A study of note by Stenson and colleagues examined the relationship between obesity and inpatient rehabilitation outcomes for patients with tSCI; it found that obese patients had longer hospital stays and less improvement in motor function when compared to non-obese patients (40). Furthermore, this same study concludes that obese patients were less likely to be discharged home and more likely to be discharged to another healthcare facility (40). Additionally, other studies found that obesity not only affects a patient’s recovery process, but also affects the required healthcare resources and rehabilitation needs of these patients (41, 42). By incorporating obesity as a baseline variable in our study, we were able to distinguish between patient groups when evaluating their hospital or rehabilitation facility LOS. Our result is consistent with previous studies reporting a relationship between obesity and prolonged LOS in patients with tSCI (40, 41).

Our sample included tSCI patients with trauma to any vertebral level from C1 to L5 and resultant impairment to the spinal cord or cauda equina (43). In the interest of finding naturally-emerging patterns in the patient sample, we did not prespecify groups based on the anatomical location of their injury. Based on our results, the PLI was among the primary factors that distinguished patient subgroups. Furthermore, when observed from an inter-group perspective, this classification characterized patients into a purely cervical spine trauma (subgroup 1), a mixed lower cervical or/and thoracic spine trauma (subgroup 2), a purely thoracic spine trauma (subgroup 3), a lower thoracic spine trauma (subgroup 4), and a lumbar spine trauma (subgroup 5).

The choice inclusion of SC in our method identified subgroups of patients with a pattern of PLI that was clinically informative; these subgroups separated traumatic injury to the spinal cord (subgroups 1, 2, 3, and 4) from trauma below the spinal cord (subgroup 5), which is more likely to produce cauda equina injury. Our findings extend on previous work examining the factors associated with traumatic cauda equina injury (tCEI), an understudied condition (44). SC identified a patient subgroup with comparable characteristics to those reported by Attabib and colleagues (44), including a similar mean age and a bimodal distribution in initial injury severity (i.e., peaks at AIS A and D). Notably, patients in subgroup 5 demonstrated the most favourable outcome across all patient subgroups, supporting the notion that these patients have a considerable chance of functional recovery after injury (44). However, it is important to note that Attabib and colleagues used the NLI as determined by the ISNCSCI to classify patients as having tCEI, whereas we opted to use PLI as the only anatomical data in this study.

Potential subjectivity in evaluating NLI may impact the accuracy of tSCI patient classification methods such as the groundwork laid by Dvorak and colleagues (45, 46). Our data-driven approach alleviates this reliance on NLI assessment and improves the Canadian Classification method in two primary ways.

First, reliability of assessment of NLI might impact the consistency of data collected for classifying tSCI patients. The Canadian classification’s singular reliance upon the neurological examination to collect baseline characteristics – NLI and injury severity based on AIS grade – have some practical drawbacks. For instance, conducting baseline examinations in an acute setting is challenging, and results can be confounded by patient-specific factors such as intoxication, sedation, or concurrent brain injuries (46–48). This represents real-world clinical challenges that contribute to the complexity of tSCI patients. To address these issues, our analysis incorporated cluster analysis using context-relevant data to explore inherent patterns within our study population and identify clinically relevant patient subgroups. Furthermore, the PLI was found to be a significant factor; a surrogate for the skeletal level of injury referring to the vertebral column level on the radiograph where trauma has taken place. This demonstrates PLI’s potential as an alternative anatomical classification of SCI that is more accurate and reliable than the ISNCSCI neurological assessment, which is susceptible to poor reliability (49).

Second, the Canadian Classification system uses *a priori* to classify patients (9). This may affect the generalizability in different clinical settings, where data inaccuracies triggered by patient-specific factors can introduce bias. This may in turn influence the applicability of the data, particularly when translating novel therapeutics from animal models to clinical practice (1). In contrast, our study sought to explore the clinical insights obtainable when context-relevant data alone drives the analysis without depending on outcome variables. We discovered patterns linking variables to outcomes that naturally incorporated data applicability. By applying SC to a set of baseline variables, we were able to uncover key interactions among demographic, anthropometric, and clinical variables such as age, body mass index, and injury mechanism/etiology.

Total LOS had high standard deviations in all subgroups comparing to other variables. This high variation within each cluster supports the finding of Craven and colleagues (24), who developed a prediction model for patients’ rehabilitation LOS in Canada. Their findings report that the prediction of rehabilitation LOS is beyond impairment characteristics (e.g., administration, resource allocation etc.). As our subgroups were created based on demographics and injury characteristics, the high variability in LOS is therefore understandable.

Although the rate of change among the subgroups from inpatient rehabilitation to discharge in FIM motor score (“FIM Motor Difference”) was not significantly different, each one showed improved scores. Subgroups 1, 3, 4, and 5 had similar mean FIM motor score changes, while subgroup 2, which had the longest LOS and the smallest FIM motor score at discharge, had the lowest mean FIM Motor Difference. This trend generates several hypotheses: first, considering the variation in different locations of injury in subgroup 2 and the lowest FIM Motor Difference, it is possible that patients in this subgroup may have suffered from multiple traumas as the majority of cases included in this group report high energy injuries. Second, while the FIM Motor Difference was not statistically different among subgroups, the similar mean value across subgroups 1, 3, 4, and 5, demonstrates their similar potential of motor gain for independence. In comparison, the complete injury in subgroup 2, which had a lower mean FIM Motor Difference, seemed to lead to limited independence gain, and therefore results in a longer hospital stay. Further investigation is required to validate these data-driven insights of FIM motor score change.

When additional injury-related variables (i.e., mechanism and energy of injury) were superimposed on the patient subgroups, they exhibited patterns of traumatic injury that were distinct and clinically intuitive (Table 4). For example, subgroup 1 – which counts comparatively more elderly patients – had the highest proportion of injuries attributed to low-energy mechanisms (76.11%), and of injuries specifically caused by falls (56.64%). In contrast, subgroups 2 to 5 had a higher proportion of high-energy mechanisms attributed to transportation, and patients in these subgroups were comparatively younger. This corresponds with the literature on the etiology of tSCI, which reports high-energy impacts (e.g., traffic accidents and sport-related injuries) as more common in younger individuals, and low-energy impacts (e.g., falls) as a more frequent occurence in older adults, who commonly have degenerative changes leading to central cord syndrome or osteoporotic fractures of the cervical spine (50, 51).

To further understand the characteristics and clinical significance of the identified subgroups, we analyzed the data from Tables 4, 5 and compared them to relevant clinical literature. Subgroup 2 predominantly consists of patients with complete spinal cord injuries (tetra/paraplegia) resulting from motor vehicle accidents (52–54). Subgroup 5 includes patients with cauda equina injury or sacral dysraphism following a spinal cord injury (44, 53, 55). Subgroup 4 comprises injuries in the thoraco-lumbar region, often burst fractures (53, 56, 57). Subgroup 3 counts more chance fractures or seat belt fractures resulting in a spinal cord injury (40, 53, 55). Finally, subgroup 1 represents low energy falls in the elderly that result in a spinal cord injury (22, 55, 58, 59).

In our study, a distinctive pattern emerged within subgroup 1, revealing an overlap of injuries across both the upper and lower cervical regions. This observation is particularly prevalent among the elderly participants whose predominant mechanism of injury was falls. This pattern accentuates the vulnerabilities of the elderly demographic to spinal injuries, consistent with established clinical findings (60). While the clinical literature broadly categorizes cervical injuries into distinct upper and lower zones, our data-driven examination highlights the nuances of injury patterns, suggesting that such traditional delineations may manifest differently within specific patient demographics.

Our findings also provide insight into the prognosis of these different patient profiles (Table 5). Older age has a negative impact on neurological and functional recovery (61). However, our analysis shows that older patients with tSCI caused by the prototypical geriatric fall (subgroup 1) have a relatively moderate prognosis when compared to younger patients in other subgroups. This suggests that age should be considered in the context of other factors, such as the energy and location of injury, and ensuing neurological deficit (15) when predicting motor impairment and LOS.

In order to assist clinicians providing care for tSCI patients and allow personalized approaches to treatment, classifications to evaluate patients need to take the heterogeneity of SCIs into consideration (9). Our analysis identified five subgroups of patients that could be described in a simple yet intuitive manner, producing patient and injury-related labels at presentation and discharge (Table 5). Furthermore, our analysis allowed an exemplar case to be drawn from each patient subgroup. We were able to illustrate that baseline clinical factors commonly available in the acute setting (age, BMI, injury-related information) can contribute to a better understanding of individual patient needs, potentially enabling more tailored care. A national survey of Canadian SCI centers revealed that insufficient SCI-specific knowledge, poor recognition of the condition in the acute setting, and communication between clinicians were all major challenges to providing specialized SCI care (29). The identification of clinically similar subgroups of tSCI patients and presenting their clinical characteristics is a step towards addressing these challenges by equipping clinicians with a way to better recognize and communicate this condition. By applying advanced machine learning algorithms to sift through greater combinations of clinical variables without *a priori* assumptions, it becomes possible to reveal previously unrecognized patterns within the analyzed data or consolidate known associations within the variables. These results could provide clinically relevant insights for clinicians managing patients with tSCI, especially as large multicenter SCI registries accumulate more data.

In the realm of healthcare, the vast potential of AI is undeniable. Similarly, the need for rigorous oversight in AI-driven research is evident (62, 63). Recognizing both the promise and the challenges, in our study, we prioritized clinical relevancy by basing our data selection on clinically accepted practices. We applied unsupervised learning to the data to unveil inherent patterns without making prior assumptions, which resulted in classifications tied to the specific attributes of the cohort. The clinical relevancy of the identified subgroups was examined through a rigorous process involving an extensive review of clinical literature and consultations with medical experts. Thus, our methodology offers a refined perspective on data-driven patient categorization, underscoring the significance of clinical relevancy in the application of AI in healthcare. This insight could promote a more tailored, patient-centric approach to care and treatment strategies.

## 5. Limitations

Our research utilized data drawn from a Canada-wide, prospectively collected registry with a limited number of patients, which may restrict generalizability. Although the variables used in the cluster analysis may differ if replicated elsewhere, the derived patient labels presented are clinically intuitive and might be generalizable across care systems.

While cluster analysis can be a useful tool in new research, it has some limitations that should be considered. Different clustering methods may identify different subgroups, which may be sensitive to dropped cases. In addition, there are limited ways to validate these obtained subgroups. In the case of this study, we used cluster analysis to demonstrate the potential of a data-driven approach to autonomously separate patient populations that are clinically distinct. However, it should be noted that the clustering method does not currently offer a straightforward way to assign a patient into a specific group as it does not use cut-off values, but rather groups patients based on their averaged similarities across the input variables.

In our pursuit to demonstrate the utility of a data-driven methodology as a supplementary approach for patient categorization, we must acknowledge the study’s findings are bound by the dataset’s scope and completeness of the respective data in our study cohort. This accentuates the need for comprehensive data to facilitate nuanced analyses in subsequent research.

## 6. Future work

Future research on tSCI can focus on improving and expanding the use of cluster analysis in databases, as well as building on its results to develop prediction models for patterns of patient recovery. Future studies could also involve focusing on specific domains of interest, such as patient outcomes, motor and sensory functioning, and could consider additional patient characteristics such as interventions and socio-demographics in the acute clinical evaluation. In addition, incorporating advanced neuroimaging and molecular biomarkers, which are more sensitive to disease processes (64), may provide insight into how these data could contribute to predicting and customizing the individual trajectories of recovery and unique needs of each patient.

## 7. Conclusion

We deployed spectral clustering method and identified five subgroups of traumatic spinal cord injury patients with clinical intra-group similarities and statistically significant inter-group differences for baseline demographic and injury characteristics collected at admission and outcome variables at discharge. This data-driven approach resulted in clinically relevant and plausible insights without depending on *a priori* decisions, a step toward better understanding of the heterogeneity inherent in tSCI. We demonstrated that cluster analysis can be used to further define the patterns or groups of other patient characteristics that exist in the tSCI population, thus contributing to categorizing the tSCI population into subgroups with distinct needs. This data-driven patient categorization holds the potential to support the delivery of more specialized, patient-centered care.

## Data availability statement

The data analyzed in this study was obtained from the Rick Hansen Spinal Cord Injury Registry (RHSCIR), managed by the Praxis Spinal Cord Institute (https://praxisinstitute.org/research-care/key-initiatives/national-sci-registry/), the following licenses/restrictions apply: access to these datasets is subject to adherence to RHSCIR Data Use and Disclosure Policy. Requests to access these datasets should be directed to the Praxis Spinal Cord Institute, dataservices@praxisinstitute.org.

## Ethics statement

The studies involving humans were approved by ethics approval was obtained from each contributing site’s Research Ethics Boards (see the acknowledgement section of the manuscript for site details). The studies were conducted in accordance with the local legislation and institutional requirements. Participants either provided written informed consent for participation in this study or had a minimal chart review dataset collected under a consent waiver.

## Author contributions

SB: Investigation, Software, Visualization, Conceptualization, Methodology, Validation, Writing – original draft, Writing – review & editing, Formal analysis. RH: Validation, Methodology, Resources, Writing – review & editing. NB: Resources, Supervision, Writing – review & editing, Funding acquisition. WM: Resources, Validation, Supervision, Writing – review & editing, Conceptualization, Funding acquisition, Methodology. HV: Validation, Resources, Supervision, Writing – review & editing. EW: Validation, Resources, Writing – review & editing. AS: Validation, Resources, Writing – review & editing. SK: Validation, Resources, Writing – review & editing. J-MM-T: Validation, Resources, Writing – review & editing. ET: Validation, Resources, Writing – review & editing. ZW: Validation, Writing – review & editing. PP: Methodology, Project administration, Validation, Conceptualization, Investigation, Funding acquisition, Supervision, Writing – review & editing.
